# Production of Bio-Oils and Biochars from Olive Stones: Application of Biochars to the Esterification of Oleic Acid

**DOI:** 10.3390/plants11010070

**Published:** 2021-12-27

**Authors:** Francisco José Sánchez-Borrego, Tomás Juan Barea de Hoyos-Limón, Juan Francisco García-Martín, Paloma Álvarez-Mateos

**Affiliations:** Departamento de Ingeniería Química, Facultad de Química, Universidad de Sevilla, 41012 Seville, Spain; fsanchez25@us.es (F.J.S.-B.); tbareadehoyoslimn@gmail.com (T.J.B.d.H.-L.)

**Keywords:** biodiesel, bio-oil, levoglucosenone, olive stones, pyrolysis

## Abstract

Olive stones are a by-product of the olive oil industry. In this work, the valorisation of olive stones through pyrolysis was attempted. Before pyrolysis, half of the samples were impregnated with sulphuric acid. Pyrolysis was carried out in a vertical tubular furnace with a ceramic support. The pyrolysis conditions assayed were: temperature between 400 and 600 °C, heating ramp between 5 and 20 °C∙min^−1^, and inert gas flow rate between 50 and 300 mL Ar∙min^−1^. Among them, temperature was the only parameter that influenced the pyrolysis product distribution. The most suitable temperature for obtaining biochar was 400 °C for both non-treated and pre-treated raw material, while for obtaining bio-oil, it was 600 °C for impregnated olive stones and 400 °C for the raw material. The impregnated olives stones led to bio-oils with much higher amounts of high-added-value products such as levoglucosenone and catechol. Finally, the biochars were impregnated with sulphuric acid and assayed as biocatalysts for the esterification of oleic acid with methanol in a stirred tank batch reactor at 60 °C for 30 min. Biochars from non-treated olive stones, which had lower specific surfaces, led to higher esterification yields (up to 96.2%).

## 1. Introduction

The agricultural exploitation of olive tree accounts for 11 × 10^6^ ha in the world, most of them in Mediterranean countries, for example, Spain, Italy, Greece, Morocco, etc. As a result, 20 × 10^6^ t [1] olives a year are produced worldwide. The main exploitation is the production of olive oil, which produces large amounts of by-products. Another main products of olives are table olives, known throughout the world as pickles and used as an ingredient in cooking.

In some olive oil companies, the olives are first de-stoned before entering the olive oil extraction system, in which the olives go through a decanter of two or three outlets to obtain olive oil and pomace (containing 65–75 wt.% moisture), or olive oil, pomace (containing 45–55 wt.% moisture), and wastewater, respectively [1,2]. This work focuses mainly on olive stones (OS), which have an estimated production of 42,900 t/year in Spain [3].

OS are made up of cellulose, hemicellulose, and lignin. Their composition of these components is different depending on the olive variety, ranging between 27.1 and 36.4 wt.% of cellulose; 24.5 and 32.2 wt.% of hemicellulose, and 23.1 and 40.4 wt.% of lignin [1,4,5,6].

One of the potential uses of OS is the production of renewable energy through their biochemical transformation into biofuels (bioethanol) [7]. Furthermore, they could be used as a lightweight aggregate in construction mortars [8], as a feed flour with high protein, fibre, and omega-3 content [9], and as a bioplastic precursor [10].

However, the most frequent use of this by-product is combustion to obtain thermal energy. Its high calorific value and low cost make olive stones an excellent lignocellulosic material for energy. Combustion generates pollution for the environment, emitting gases such as CO_2_, CO, SO, SO_2_, and polycyclic aromatic hydrocarbons. There are alternative processes, less harmful in their use, for specific other thermochemical treatments such as pyrolysis, torrefaction, and gasification [11].

Pyrolysis is a thermochemical process carried out at high temperatures, between 400 and 700 °C, under an inert atmosphere (total absence of oxygen). During the pyrolysis process, each lignocellulose material undergoes different reaction mechanisms (that is, decarboxylation, dehydration, and demethylation) resulting in the production of bio-oil, syngas, and biochar [12].

In addition to the characteristics of the raw material used, there are several factors that influence the process, namely temperature, heating ramp, and gas flow rate [13]. It is important to note the effect of temperature on obtaining of pyrolysis products, since higher percentages of bio-oil are obtained at high temperatures (500–700 °C), while a higher proportion of biochar is obtained at low temperatures (350–400 °C) [14].

Pyrolysis can be fast or slow, depending on the heating ramp and residence time. Lower process temperatures and heating ramps and longer gas residence times improve biochar production. Approximately 35% of the weight of dry biomass can be turned into biochar, although higher pressure can provide significantly higher performance [15].

Bio-oil is a mixture of organic compounds such as esters, acids, and aromatic compounds depending on the composition of the raw material; thus, research has been carried out in recent years on the use of these components. One of these applications is the extraction of some aromatic components, such as phenols, and other compounds of high added value such as levoglucosenone or catechol. Furthermore, it is important to mention that bio-oil is also used for the manufacture of high-quality biofuels for internal combustion engines [16]. However, no information can be found in the available literature on the composition of bio-oils from olive stones.

Non-condensable gases (syngas) are a mixture of basic components such as carbon monoxide (CO) and hydrogen (H_2_). The higher heating value of syngas (4.37–5.68 MJ/m^3^) plays an important role in the generation of energy in cogeneration plants [17].

Biochar is a non-volatile carbon-rich solid residue composed of the non-hydrocarbon residues of biomass, mainly parts of lignin, oxides (usually metallic), and heavy metals, depending on the composition of the feedstock. Among all the applications that biochar currently has, its use as a catalyst can be highlighted, for example, in the esterification reaction of oleic acid with methanol to obtain biodiesel [18,19]. Many researchers have found that biochar can be used as an alternative adsorbent to remove different types of pollutants, such as heavy metals, nutrients, and pharmaceuticals, from aqueous solutions [20]. Many raw materials and their resulting biochars have low specific surface or low catalytic activity, so these materials are pre-treated (activated) with acids, such as sulphuric acid [21] or phosphoric acid [22]. To the best of our knowledge, biochars from olives stones have not been applied as biocatalyst so far.

Based on all the considerations mentioned above, the objectives of this work were as follows:

- Valorise olive stones through pyrolysis;
- Assess the most suitable pyrolysis conditions for the production of biochar and bio-oil;
- Characterize the biochars and bio-oils obtained;
- Establish the most suitable conditions for obtaining levoglucosenone and catechol in bio-oils;
- Apply the biochar obtained under the most suitable pyrolysis conditions as a biocatalyst for the esterification reaction of free fatty acids with methanol to obtain biodiesel.

## 2. Results

### 2.1. Characterisation of the Raw Material

#### 2.1.1. Thermogravimetric Analysis (TGA)

The OS were analysed by thermogravimetry using the thermobalance under the conditions described in Section 3.4.1 in order to study their decomposition and obtain information on the optimum carbonisation temperature.

**Figure 1:**
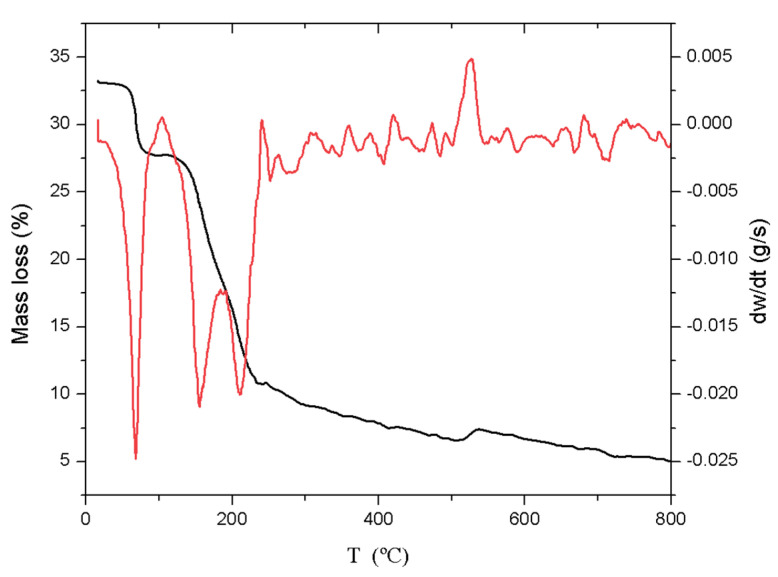
TG (black line) and DTG (red line) curves obtained from the carbonisation of OS.

Figure 1 shows the different peaks corresponding to the mass loss during heating. Mass loss at temperatures around 100 °C was associated with moisture content. In this case, the loss occurred around 70 °C, with a total loss of 15 wt.%, corresponding to physically adsorbed water that interacts only with other water molecules, a band that extended up to 120 °C [23].

Cellulose polymers degraded between 150 and 200 °C [24], and in this case showed a maximum degradation rate at 165 °C, as observed in the peak of the DTG curve. This loss was 24% of the total weight of the sample.

On the other hand, degradation of hemicellulose was observed from 200 to 250 °C [24], accounting for 27 wt.% of the sample.

In Figure 1, lignin did not show a well-defined degradation peak. On the contrary, the degradation process started at 200 °C and continued gradually up to 800 °C, a temperature at which more than 80 wt.% of the initial mass had been decomposed [24], corresponding to approximately 15 wt.% of the total weight of the raw material.

As can be seen in the TGA, a large part of the weight loss (70%) had already occurred at 400 °C (Figure 1), which indicated that the sample consisted mainly of water, cellulose, and hemicellulose. It was also observed that 80 wt.% degradation of the initial mass was reached at 600 °C. Therefore, either of the two maximum temperatures assayed in the pyrolysis process ensured the carbonisation of most of the olive stones.

Finally, at 800 °C, there was still around 20 wt.% of the olive stones undegraded by pyrolysis, which corresponds to carbon residues and metallic compounds.

When comparing these data with previous work carried out with OS [25], it could be seen that the product yield was very similar. Cellulose and lignin yields were lower (24.0 vs. 26.8 wt%. and 15.0 vs. 20.0 wt.%, respectively), but that of hemicellulose was higher (27.0 vs. 25.5 wt.%).

#### 2.1.2. FTIR Analysis

**Figure 2:**
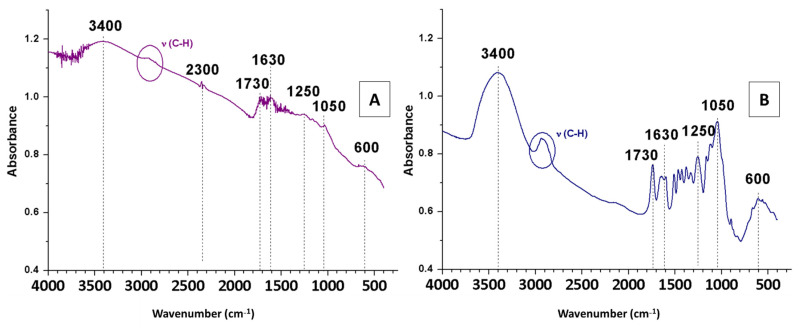
FTIR spectra of OS + H_2_SO_4_ (**A**) and OS (**B**).

The FTIR spectra of OS pre-treated with sulphuric acid (OS + H_2_SO_4_) were compared with those of the non-treated raw material (OS), with the purpose of evaluating the influence of this pre-treatment on the structure (Figure 2).

The spectrum of OS + H_2_SO_4_ (Figure 2A) showed differences in most bands compared to the FTIR spectrum or the raw material (Figure 2B). This was explained by the fact that sulphuric acid degraded hemicellulose and cellulose, which could be seen in the 3500–3100 cm^−1^ bands corresponding to OH vibrations (due to the presence of cellulose and hemicellulose with their abundant alcoholic hydroxyl groups or the symmetric and asymmetric stretching vibrations related to H_2_O molecules) and in the aliphatic bands around 2900 cm^−1^. The band at 2300 cm^−1^ corresponded to the asymmetric tension vibration of the CO_2_ molecule, which was higher in intensity in the spectrum of OS + H_2_SO_4_ compared to that of OS. Regarding the band at 1730 cm^−1^, it had a lower intensity with pre-treatment (Figure 2A) than without pre-treatment (Figure 2B), due to the degradation of hemicellulose by sulphuric acid. Furthermore, sulphuric acid degraded not only hemicellulose and cellulose, but also lignin. Although lignin degradation was lower, it can be seen reflected in the C=C bands (at 1630 cm^−1^), which are characteristic of lignin aromatics [23]. Since the bands at 1250 and 1050 cm^−1^ were related to glycosidic bonds (found mainly in hemicellulose and cellulose), their intensity was greatly reduced after treatment. Finally, the band at 600 cm^−1^, characteristic of calcium, remained completely unchanged.

### 2.2. Pyrolysis Yields

Table 1 shows the yield of each product obtained in the different pyrolysis processes according to the operational conditions.

Regarding the pyrolysis temperature, it was inversely proportional to the biochar production (Table 1), but directly proportional to the fluent products (biogas and bio-oil) [26].

Regarding the heating ramp, pyrolysis from OS + H_2_SO_4_ led to the same biochar yield. However, modifying the heating ramp achieved a reduction in the pyrolysis process time while leading to the same biochar yield, that is, the use of a heating ramp of 20 °C∙min^−1^ resulted in time savings of 20 min and 1 h when compared to the use of heating ramps of 10 and 5 °C∙min^−1^, respectively. In addition, the reduction of the pyrolysis process time also resulted in energy savings, with 20 °C∙min^−1^ being the optimal heating ramp. Similarly, the resulting percentages of biochar were again similar in the pyrolysis of OS. However, the influence on bio-oil should be highlighted, because the lower the heating ramp, the higher the bio-oil yield. Therefore, 5 °C∙min^−1^ is the optimal heating ramp when the objective is to obtain bio-oil.

Furthermore, in terms of argon flow rate, the pyrolysis of OS + H_2_SO_4_ under 150 mL Ar∙min^−1^ produced the highest amounts of biochar and bio-oil. When OS was used as raw material (without sulphuric acid pre-treatment), there were hardly any differences in the yields of pyrolysis products when the argon flow rate was varying. Pyrolysis under 50 mL Ar∙min^−1^ should be highlighted, as it was the one that achieved the highest biochar and bio-oil yields from non-treated OS, as well as the lowest Ar expenditure.

Finally, in light of these data (Table 1), it could be said that feedstock that was previously pre-treated (OS + H_2_SO_4_) led to higher biochar yields. These higher biochar yields are due to the formation of chemical bonds between two polymer chains (cross-link) and the retention of low-molecular-weight carbonaceous species in the solid phase, stabilising the cellulose structure [27]. The results showed that acid pre-treatment resulted in 60% of biochar yield, compared to only about 31% of biochar yield without pre-treatment [28].

Pyrolyses 1 and 9 and Pyrolyses 3 and 6 were performed under the same conditions, respectively (Table 1). However, the product yields showed some differences even though the OS were from the same batch. This is because two pre-treatments were performed at different times during the execution of the experiments in this research. The first involved the first to the fifth samples, while the second involved from the sixth to the ninth samples.

These differences were based on the sulphuric actuation time (4 vs. 8 min) in the pre-treatment with sulphuric acid. The longer the residence time, the greater the retention of carbonaceous biomass, which had a direct influence on product yields (Table 1), as the longer the contact time with H_2_SO_4_, the higher the biochar yield [27].

### 2.3. Bio-Oil Characterisation

The bio-oils were subjected to gas chromatography–mass spectroscopy analysis in order to obtain a qualitative and semi-quantitative analysis of the major compounds. The samples selected were those from Pyrolyses 3 and 12, as they were obtained from pyrolysis under the same conditions, but with different raw material. Specifically, the bio-oil obtained from Pyrolysis 3 was obtained from OS + H_2_SO_4_ and that from Pyrolysis 12 was from OS. Table 2 illustrates the compounds identified by GC–MS, with a probability of matching with the database greater than 40%.

The influence of OS pre-treatment on the bio-oil composition can be observed in Table 2. Some of the compounds found in the bio-oil produced from OS + H_2_SO_4_ are more valuable than those obtained from OS, such as levoglucosenone, catechol, or vanillin. This could be explained by the transformation of lignocellulosic compounds into levoglucosenone under acidic conditions [29,30,31].

Levoglucosenone was one of the most abundant compounds in the bio-oil obtained from the pyrolysis of pre-treated OS, accounting for almost 10% of the bio-oil. This compound has a high market value: 10 mg of levoglucosenone (95%) as a laboratory reagent currently cost 165 euros (Sigma Aldrich, St Louis, MO, USA, 11/2021).

### 2.4. Biochar Characterisation

FTIR spectroscopy was used for the analysis of the surface functional groups in the biochars obtained under different conditions (acid pre-treatment, temperature, heating ramp, and argon flow rate).

#### 2.4.1. Temperature Influence

**Figure 3:**
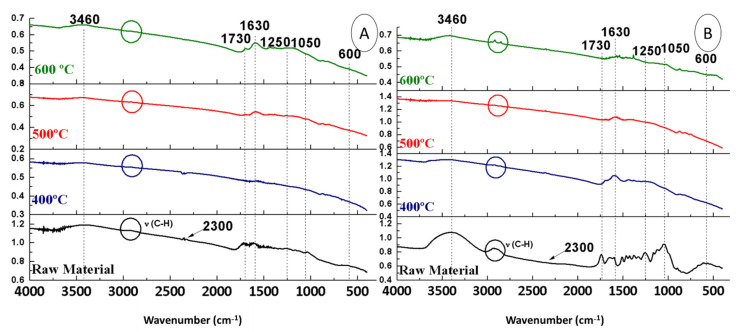
FTIR spectra of biochars from OS + H_2_SO_4_ (**A**) and OS (**B**) at different temperatures.

In order to assess the effect of temperature, the FTIR spectra of the raw materials (OS + H_2_SO_4_ and OS) were compared with those of the biochars obtained from them at different maximum pyrolysis temperatures, keeping the rest of the conditions constant (150 mL Ar∙min^−1^ and 10 °C∙min^−1^) (Figure 3).

The first band observed in both Figure 3A,B was at 3400 cm^−1^. This is usually associated with water absorption (OH vibration), which is explained by the fact that the analysis was carried out under ambient conditions (with humidity in the atmosphere).

Regarding the OS + H_2_SO_4_ biochars (Figure 3A), it could be seen that at 600 °C there were no traces of aliphatic hydrocarbons (2900 cm^−1^). The band at 2300 cm^−1^ (CO_2_) appeared only at 400 °C. On the contrary, the bands at 1730 cm^−1^ (C=O) and 1630 cm^−1^ (C=C) remained constant and only disappeared when pyrolysed at 400 °C. The peaks observed at 1250 and 1050 cm^−1^, related to CO in glycosidic bonds, were inversely proportional to temperature. The aromatic compounds, which appeared at 700–900 cm^−1^, did not seem affected by the temperature. Finally, the peak at 600 cm^−1^ (Ca) disappeared completely in the FTIR spectra of biochars from OS + H_2_SO_4_ (Figure 3A).

Regarding the OS biochars (Figure 3B), the band at 2900 cm^−1^ was only appreciated at the temperature of 600 °C. The band at 2300 cm^−1^ (CO_2_) was barely appreciated after pyrolysis. When the raw material is pyrolysed at 600 cm^−1^, the band at 1730 cm^−1^ (C=O) and at 1630 cm^−1^ (C=C) disappeared completely. While the one at 1250 cm^−1^ was not influenced by the temperature, the one at 1050 cm^−1^ was directly proportional to the temperature. In addition, aromatic compounds were present in all biochars (region 700–900 cm^−1^), even at 600 °C. Finally, the peak at 600 cm^−1^ completely disappeared in all experiments after the pyrolytic treatment.

#### 2.4.2. Influence of the Heating Ramp

**Figure 4:**
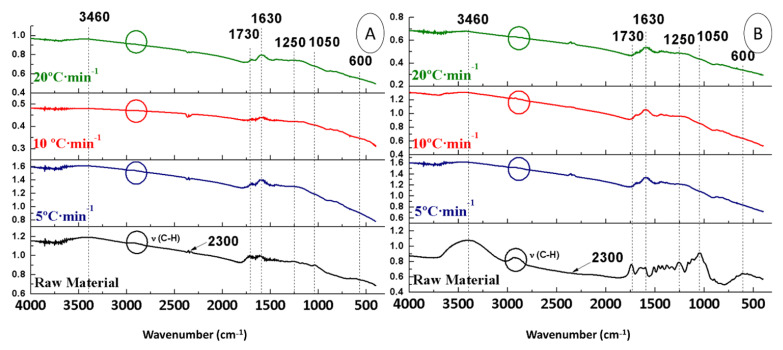
FTIR spectra of biochars from OS + H_2_SO_4_ (**A**) and OS (**B**) at different heating ramps.

To assess the effect of the heating ramp, the FTIR spectra of the raw materials (OS + H_2_SO_4_ and OS) were compared with those of the biochars obtained from them at different heating ramps, keeping the rest of the conditions constant (400 °C and 150 mL Ar∙min^−1^) (Figure 4).

Regarding the OS + H_2_SO_4_ biochars (Figure 4A), it could be seen that the absorbance band in the 3400 cm^−1^ region was still present due to absorption of humidity. However, the aliphatic band (2900 cm^−1^) was not present in the samples. The band at 2300 cm^−1^ (CO_2_) was kept stable in all samples. The bands at 1730 cm^−1^ (C=O) and at 1630 cm^−1^ (C=C) still appeared, being lower at 10 °C∙min^−1^. The peaks observed at 1250 and 1050 cm^−1^ (CO) disappeared after pyrolysis, so both bands did not rely on the heating ramp. The band at 700–900 cm^−1^ was kept stable in all the samples. Finally, the peak at 600 cm^−1^ (Ca) did not change with the heating ramp.

As for OS biochars (Figure 4B), the band at 3400 cm^−1^ (H_2_O) remained in all samples. On the contrary, the band at 2900 cm^−1^ was not present in the samples. The band at 2300 cm^−1^ (CO_2_) remained stable in all samples. The band at 1730 cm^−1^ (C=O) and at 1630 cm^−1^ (C=C) remained constant, being lower at 20 °C∙min^−1^. The bands at 1250 and 1050 cm^−1^ (CO) disappeared after pyrolysis. There was no influence of the heating ramp on the band at 700–900 cm^−1^. Finally, the peak at 600 cm^−1^ (Ca) disappeared completely after pyrolysis.

#### 2.4.3. Argon Flow Influence

**Figure 5:**
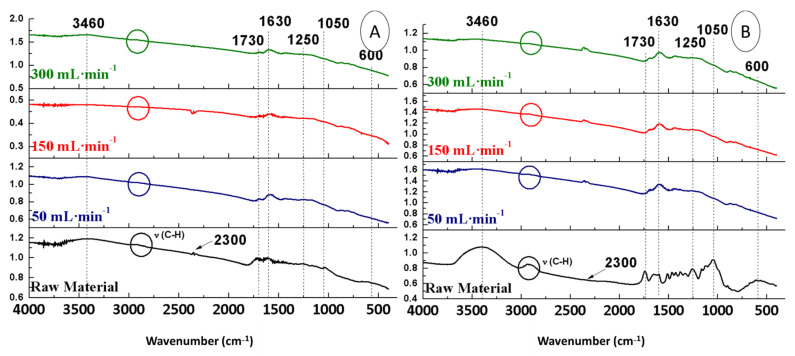
FTIR spectra of biochars from OS + H_2_SO_4_ (**A**) and OS (**B**) at different Ar flows.

To assess the effect of the argon flow during pyrolysis, the FTIR spectra of the raw materials (OS + H_2_SO_4_ and OS) were compared with those of the biochars obtained at different argon flows, keeping the rest of conditions constant (400 °C and 20 °C∙min^−1^ (to OS + H_2_SO_4_) and 5 °C∙min^−1^ (to OS)) (Figure 5).

Regarding the OS + H_2_SO_4_ biochars (Figure 5A), it could be seen that the 3400 cm^−1^ band was still present due to the absorption of water from the atmosphere. However, aliphatic hydrocarbons (2900 cm^−1^) were not present in the samples. The band at 2300 cm^−1^ (CO_2_) only appeared at 150 mL Ar∙min^−1^. The bands at 1730 cm^−1^ (C=O) and at 1630 cm^−1^ (C=C) still appeared, being lower at 150 mL Ar∙min^−1^. The peaks observed at 1250 and 1050 cm^−1^ (CO) disappeared after pyrolysis. Aromatic compounds (800 cm^−1^) did not appear to be affected by argon flow. Finally, the peak at 600 cm^−1^ due to Ca disappeared completely.

Finally, with respect to the OS biochars (Figure 5B), the band at 3400 cm^−1^ (H_2_O) remained in all samples. On the contrary, the band at 2900 cm^−1^ was not present in the samples. The band at 2300 cm^−1^ (CO_2_) remained stable. The band at 1730 cm^−1^ (C=O) and at 1630 cm^−1^ (C=C) still occurred, keeping stable in all samples. The bands at 1250 and 1050 cm^−1^ (CO) disappeared after pyrolysis. Argon flow did not influence aromatic compounds (800 cm^−1^). Finally, the characteristic peak of calcium (600 cm^−1^) disappeared completely.

In summary, it could be concluded that the most influential parameter on biochar spectra was the maximum pyrolysis temperature, which is reflected in Section 2.4.1.

#### 2.4.4. Specific Surface of Biochars

The specific surface area is an important parameter, as it could influence the use of biochars from OS as a catalyst for esterification reactions. The specific surface area of the biochars obtained under the different pyrolysis conditions is illustrated in Table 3.

It could be observed that the sulphuric pre-treatment of OS increased the specific surface area in biochars. Therefore, all biochars obtained from pyrolysis of OS + H_2_SO_4_ had higher specific surface area than those obtained from OS without pre-treatment under the same conditions. Therefore, the highest BET surface was obtained at 600 °C with OS + H_2_SO_4_ as a raw material.

### 2.5. Application of Biochars as Biocatalyst for the Esterification Reaction

The acid index of the commercial oleic acid was 182.18 mg KOH/g oil. The acid index was used to follow the reaction. Two phases were obtained: the organic phase (upper phase) composed of methyl esters, and the aqueous phase (lower phase). Table 4 shows the percentage of both phases in each esterification reaction carried out with the different biochars obtained from pyrolysis (activated and non-activated).

The esterification yields obtained were similar to those found in the literature using biochars from the pyrolysis of *Jatropha curcas* L. [18] and microalgae [19], whose authors reported esterification yields of up to 83.6 and 94.2%, respectively.

The esterifications using the biochars from OS, which had low specific surfaces (Table 3), provided better yields than when using the biochars from OS + H_2_SO_4_ (Table 4). This could be explained by the fact that the lower the specific surface, the larger the pore size. The larger the pore size, the better the absorption of large molecules such as sulphuric acid [32]. Furthermore, the pyrolysis temperature at which the biochar was obtained seemed to influence the esterification reaction, with 400 °C being the optimal temperature to obtain biochars for the esterification reaction.

The organic phase of Experiment 12, which achieved the highest esterification yield (96.2%), was analysed as described in Section 3.5.2. In Table 5, the compounds with a probability of matching with the database greater than 40% were identified, along with their relative area.

## 3. Materials and Methods

### 3.1. Raw Materials

The OS were provided by the Cooperativa Agrícola Olivarera Virgen del Campo S.C.A. (Cañete de las Torres, Córdoba, Spain). The reagents used were sulphuric acid (H_2_SO_4_) 98.0 wt.% (PanReac, Barcelona, Spain), methanol (CH_3_OH) 99.5 wt.% (PanReac, Barcelona, Spain), and oleic acid 65.0–88.0 wt.% (PanReac, Barcelona, Spain). The inert gas used in the pyrolysis was argon (Ar) (Al Air Liquid España, Madrid, Spain).

#### 3.1.1. Pre-treatment of Olive Stones

The OS grinding was carried out in a planetary ball mill (Retsch PM 200, Düsseldorf, Germany). The mill was programmed at 500 rpm for 20 min. Grinding was carried out to obtain an OS particle diameter size of less than 150 μm.

Once all OS were homogenized, a fraction was treated before pyrolysis with H_2_SO_4_. The pre-treatment consisted of pouring 1 mL of 98 wt.% H_2_SO_4_ per gram of OS, after which it was homogenised and washed with excess distilled water until it had neutral pH and dried in an oven at 45 °C for 24 h to remove moisture. Finally, the mixture was homogenised in a mortar.

### 3.2. Pyrolysis

**Figure 6:**
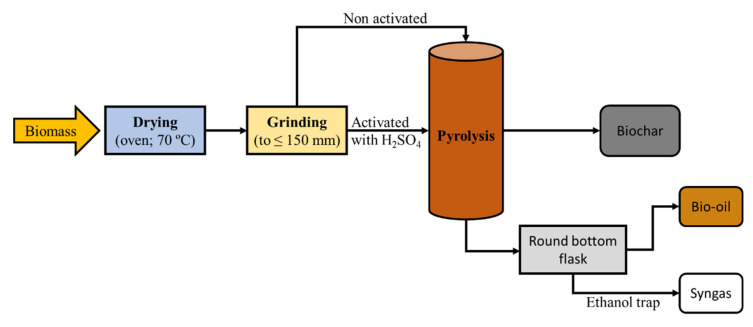
Process scheme for biomass pyrolysis.

The OS pre-treated with sulphuric acid and the OS without previous treatment were subjected to pyrolysis under an inert gas atmosphere (Ar) (Figure 6). The pyrolytic furnace (16 cm height, 3.5 cm diameter) was a vertical tubular furnace with a ceramic support with a pore size of 3 mm. Its maximum temperature is 900 °C and it is controlled by a CN300-P self-tuning PID controller (Conatec, Irún, Spain).

Between 5 g of OS pre-treated with H_2_SO_4_ or 6 g of OS untreated were introduced into the reactor. The temperatures to which the samples were subjected for 2 h were 400, 500, and 600 °C, with heating ramps of 5, 10, and 20 °C∙min^−1^. Argon flow rates of 50, 150, and 300 mL∙min^−1^ were used during the experiments (Table 6).

### 3.3. Bio-Oil Characterisation

The liquid phase was recovered by transferring it from the two-neck flask of the pyrolytic equipment to a previously tared round bottom flask,. After that, to remove the remaining residue from the bottom of the two-neck flask, a few microliters of acetone were introduced and poured into the round bottom flask. Subsequently, bio-oil was taken to a rotary evaporator (Heidolph, Schwabach, Germany) to evaporate the acetone and obtain pure bio-oil.

A qualitative analysis of the bio-oil composition from pyrolysis was performed with a TSQ8000 mass spectrometer (Thermo Fisher Scientific, Waltham, MA, USA) coupled to a triple quadrupole gas chromatograph (GC–MS) equipped with an autosampler. A Zebron ZB-5MS (Phenomenex, CA, USA) column (30 m × 0.25 mm × 0.25 µm; 5% phenylarylene, 95% dimethylpolysiloxane) and N_2_ as carrier gas were used. The analyses were carried out under the following conditions: 50 °C initial temperature, 7 °C·min^−1^ heating ramp for 30 min, and final temperature 310 °C.

The identification of the peaks was performed using the database of the Quan Browser tool of the XCalibur software (Thermo Fisher Scientific, Waltham, MA, USA). To limit the number of compounds, several factors were taken into account: relative area of the compound greater than 1, match probability of the compound in the database greater than 40%, and occurrence of the compound in a greater number of samples.

### 3.4. Biochar Characterisation

#### 3.4.1. Thermogravimetric Analysis

The thermogravimeter with which this analysis was carried out was a SDT Q600 electrobalance (TA Instruments, Inc., New Castle, DE, USA), which has a sensitivity of +/−0.5 µg. The system responsible for the generation of heat was a vertical furnace that allows a maximum pyrolysis temperature of 1200 °C to be reached.

The raw material (OS) was treated at a heating ramp of 6 °C∙min^−1^ up to 800 °C, with a constant flow of 100 mL Ar∙min^−1^. This treatment was carried out until the complete decomposition of the organic matter, which was then burned in air, obtaining only inorganic ash.

#### 3.4.2. Fourier-Transform Infrared Spectroscopy (FTIR)

A Nicolet TM iSTM5 FTIR Fourier transform infrared spectrometer (Thermo Fisher Scientific, Waltham, MA, USA) was used to characterise the feedstock and all biochars. This homogenised feedstock was compacted in a uniaxial press under pressure.

For transmission measurements, pellets were prepared using potassium bromide (KBr) as diluent, with a final sample concentration of 5 wt.%, and spectra were recorded in the 500–4000 cm^−1^ wavenumber range.

#### 3.4.3. BET Specific Surface

The BET method was used to calculate the specific surface area (SBET). A Gemini V-2365//V.1.00 adsorption equipment (Micromeritics Instrument Corporation, Norcross, GA, USA) was used. The biochars were previously degassed at 150 °C for 2 h under a N_2_ atmosphere.

### 3.5. Esterification Reaction

The esterification reactions of olive stones, whether pre-treated (impregnated) with H_2_SO_4_ or not, were carried out in a stirred tank batch reactor (25 cm height, 12 cm diameter), equipped with a temperature regulator and a gas condenser (Liebeg refrigerant cooled with water at room temperature) at 60 °C for 30 min.

The mixture was heated at 60 °C for 30 min by means of a Fibroman-C heating mantle (J.P. SELECTA, Barcelona, Spain) connected to a temperature controller. Two additional openings were used to extract the sample and introduce the thermocouple, which continuously measured the temperature.

Before esterification, all biochars, except biochars obtained from Pyrolyses 1, 2, and 6 (Table 6), were subjected to activation with sulphuric acid. Similar to the pre-treatment of the raw material (Section 3.1.1), the activation of the biochars was carried out by impregnation of 1 g of biochar with 1 mL of sulphuric acid (98 wt.%). The conditions under which the esterifications were carried out were: 10 g oleic acid, 17.2 g methanol (1:15 oleic acid to methanol molar ratio), and 1 g biochar. These conditions were selected from previous studies available in the literature [33].

Finally, the interesting products were obtained in the organic phase, which was subjected to two determinations. The first was the measurement of its acid index, and the second was the determination of the fatty acid methyl esters (FAME) by gas chromatography–mass spectroscopy in the TSQ8000 mass spectrometer (Thermo Fisher Scientific, Waltham, MA, USA) described in Section 3.5.1 and Section 3.5.2.

#### 3.5.1. Acid Index

Before the esterification started, it was important to know the acid index of the oleic acid, as the reduction of its value indicates the extent of the esterification reaction and allows the calculation of its yield. UNE-EN 14104:2003 was followed to determine the acidity of oleic acid and the esterification product. A detailed description of this procedure can be found in [34].

#### 3.5.2. Gas Chromatography-Mass Spectroscopy

The percentage of different FAME in the samples was determined by gas chromatography using methyl heptadecanoate as the internal standard. A HP 5890 series II gas chromatograph (Hewlett Packard, Palo Alto, CA, USA) equipped with a SP2380 capillary column (Sigma-Aldrich, St Louis, MO, USA) [60 m × 0.25 mm internal diameter × 0.25 μm film thickness] was used. The column temperature was set at 185 °C and then the temperature program was increased to 220 °C with a heating ramp of 3 °C min^−1^. The injection was operated in splitless mode, with injector and detector temperatures of 210 °C and 250 °C, respectively. The FAMEs were identified by mass spectrometry by comparing the spectra with those of the database for this type of compound (Wiley, NIST).

## 4. Conclusions

Thermogravimetric analysis showed that OS have a low percentage of lignin. The FTIR spectrum of the OS + H_2_SO_4_ feedstock did not show differences in spectral bands compared to that of the OS feedstock, but did show a much lower intensity of the bands. The most suitable conditions to obtain the highest biochar yield from OS + H_2_SO_4_ were 400 °C, 20 °C∙min^−1^ and 150 mL Ar∙min^−1^.The optimal conditions to obtain the highest bio-oil yield from OS were 400 °C, 5 °C∙min^−1^ and 50 mL Ar∙min^−1^.The highest specific surface area (approximately 400 m^2^/g) was obtained in the biochar from the pyrolysis at 600 °C from OS + H_2_SO_4_. The FTIR spectra of the biochars obtained from OS + H_2_SO_4_ and OS did not show noticeable differences. However, increasing the pyrolysis temperature decreases the intensity of the bands. The use of biochar from OS as catalyst in the esterification reaction showed better yield compared to the biochar from OS + H_2_SO_4_. The pyrolysis conditions in terms of biochar characteristics did not affect the esterification yield.

## Figures and Tables

**Table 1:** Biochar, syngas, and bio-oil yields obtained under different pyrolysis conditions.

Pyrolysis	Raw Material	SAT (min)	T (°C)	H_ramp_ (°C∙min^−1^)	Ar Flow (mL∙min^−1^)	Y_Biochar_ (wt.%)	Y_syngas_ (wt.%)	Y_Bio-oil_ (wt.%)
1	OS + H_2_SO_4_	4	600	10	150	44.6	38.2	17.1
2	OS + H_2_SO_4_	4	500	10	150	51.2	35.2	13.5
3	OS + H_2_SO_4_	4	400	10	150	57.8	35.6	6.7
4	OS + H_2_SO_4_	4	400	5	150	59.6	33.4	7.0
5	OS + H_2_SO_4_	4	400	20	150	60.2	31.6	8.2
6	OS + H_2_SO_4_	8	400	10	150	60.0	33.0	7.0
7	OS + H_2_SO_4_	8	400	20	300	57.2	41.2	1.6
8	OS + H_2_SO_4_	8	400	20	50	57.8	37.3	4.9
9	OS + H_2_SO_4_	8	600	10	150	50.0	47.4	2.6
10	OS	-	600	10	150	27.2	52.6	20.3
11	OS	-	500	10	150	27.7	48.8	23.5
12	OS	-	400	10	150	31.2	45.5	23.4
13	OS	-	400	20	150	29.3	40.5	30.2
14	OS	-	400	20	50	29.7	29.5	40.8
15	OS	-	400	5	150	31.7	36.2	32.2
16	OS	-	400	5	50	33.5	30.3	36.2
17	OS	-	400	5	300	30.3	44.3	25.3

**Table 2:** Bio-oils composition.

	Bio-Oils Samples	
	Pyrolysis 3	Pyrolysis 12
Compound	Area (%)	
Toluene	1.18	-
Furfural	5.30	3.80
4-Hydroxy-4-methylpentan-2-one	2.53	-
2-Furanmethanol	-	3.49
Ethylbenzene	3.03	2.34
o-Xylene	6.26	3.40
p-Xylene	2.74	1.51
Cyclopenta-1,2-dione	1.15	2.42
5-Methyl-2-furancarboxaldehyde	2.15	1.61
Phenol	3.60	-
N-Butyl-tert-butylamine	-	2.59
3-Methylcyclopentan-1,2-dione	-	2.60
2-Methylphenol	1.55	1.20
3-Methylphenol	3.53	-
2-Methoxyphenol	5.28	7.87
Levoglucosenone	9.57	-
Catechol	6.17	3.33
Creosol	6.94	7.32
1,4:3,6-Dianhydro-α-D-glucopyranose	3.51	-
3-Methylbenzene-1,2-diol	1.71	1.27
3-Methoxybenzene-1,2-diol	2.35	3.28
4-Ethyl-2-methoxyphenol	2.14	5.02
4-Methylbenzene-1,2-diol	1.59	1.55
2-Methoxy-4-vinylphenol	5.38	14.30
Eugenol	-	2.00
Vanillin	3.65	1.50
3,5-Dimethoxy-4-hydroxytoluene	6.07	5.70
Trans-isoeugenol	-	5.75
1,6-Anhydro-β-D-glucopyranose	2.88	1.51
5-Tert-butylpyrogallol	1.73	-
1-(4-Hydroxy-3-methoxyphenyl)-propan-2-one	1.52	2.02
4-Ethanoyl-2,6-dimethoxy-phenol	-	3.56
Butyrovanilone	3.15	-
4-Hydroxy-3,5-dimethoxy-benzaldehyde	1.73	-
(E)-2,6-Dimetoxi-4-(prop-1-en-1-il)-fenol	-	4.56
Coniferyl aldehyde	-	2.15
Syringylacetone	-	2.37
Butylsyringone	1.64	-

**Table 3:** Specific surface of the biochars obtained under different pyrolysis conditions.

Raw Material	T (°C)	H_ramp_ (°C∙min^−1^)	Ar Flow Rate (mL∙min^−1^)	S_BET_ (m^2^∙g^−1^)
OS + H_2_SO_4_	600	10	150	418.60
OS + H_2_SO_4_	500	10	150	263.38
OS + H_2_SO_4_	400	10	150	5.80
OS + H_2_SO_4_	400	5	150	13.75
OS + H_2_SO_4_	400	20	150	21.50
OS + H_2_SO_4_	400	10	150	42.09
OS + H_2_SO_4_	400	20	300	106.11
OS + H_2_SO_4_	400	20	50	52.95
OS + H_2_SO_4_	600	10	150	367.93
OS	600	10	150	242.70
OS	500	10	150	230.40
OS	400	10	150	4.55
OS	400	20	150	8.18
OS	400	20	50	3.94
OS	400	5	150	8.90
OS	400	5	50	6.57
OS	400	5	300	12.37

**Table 4:** Resulting acid index, esterification yield, and percentages of organic (OP) and aqueous (AP) phases obtained after esterification.

Biochar	Impregnation	Acid Index	Esterification Yield	OP (%)	AP (%)
1	No	172.2	5.5	-	-
2	No	180.7	0.3	-	-
4	Yes	13.1	92.8	63.9	32.1
5	Yes	16.1	91.2	64.1	22.8
6	No	183.7	0.0	-	-
6	Yes	26.7	85.3	52.1	26.9
7	Yes	17.4	90.4	60.8	36.8
8	Yes	12.9	92.9	58.5	26.5
9	Yes	8.8	95.2	61.0	28.5
10	Yes	9.8	94.6	62.8	23.7
11	Yes	7.1	96.1	55.3	25.3
12	Yes	7.0	96.2	63.7	26.6
13	Yes	10.3	94.3	57.7	23.0
15	Yes	8.7	95.2	49.2	28.9
16	Yes	8.4	95.4	67.3	24.8
17	Yes	7.8	95.7	59.9	25.7

**Table 5:** Composition of the organic phase obtained after esterification of commercial oleic acid.

Area (%)	Compound
5.65	Dodecanoic acid methyl ester
2.78	Methyl myristoleate
9.49	Palmitic acid methyl ester
12.79	Palmitoleic acid methyl ester
5.83	Cis-10-heptadecenoic acid methyl ester
48.78	Oleic acid methyl ester
13.50	Linoleic acid methyl ester
1.18	11-Eicosenoic acid methyl ester

**Table 6:** Pyrolysis conditions for olive stones (OS).

Pyrolysis	Raw Material	T (°C)	H_ramp_ (°C∙min^−1^)	Ar Flow (mL∙min^−1^)
1	OS + H_2_SO_4_	600	10	150
2	OS + H_2_SO_4_	500	10	150
3	OS + H_2_SO_4_	400	10	150
4	OS + H_2_SO_4_	400	5	150
5	OS + H_2_SO_4_	400	20	150
6	OS + H_2_SO_4_	400	10	150
7	OS + H_2_SO_4_	400	20	300
8	OS + H_2_SO_4_	400	20	50
9	OS + H_2_SO_4_	600	10	150
10	OS	600	10	150
11	OS	500	10	150
12	OS	400	10	150
13	OS	400	20	150
14	OS	400	20	50
15	OS	400	5	150
16	OS	400	5	50
17	OS	400	5	300

## Data Availability

The data presented in this study are available on request from the corresponding author. The data are not publicly available due to confidentiality agreements with the funding companies.
